# Burden of albinism: development and validation of a burden assessment tool

**DOI:** 10.1186/s13023-018-0894-3

**Published:** 2018-09-18

**Authors:** Fanny Morice-Picard, Charles Taïeb, Aurelie Marti, Antoine Gliksohn, Mohammed Bennani, Christine Bodemer, Khaled Ezzedine, Filière Maladies Rares en Dermatologie: FIMARAD

**Affiliations:** 1 CHU Bordeaux, Bordeaux, France; 2 FIMARAD, Hôpital Necker-Enfants Malades, APHP, Paris, France; 3 Genespoir, Paris, France; 4 Qualees, Paris, France; 5 MAGEC, CHU Necker-Enfants Malades, APHP, Paris, France; 6 Annimatrice, FIMARAD, Hôpital Necker-Enfants Malades, APHP, Paris, France; 7 EA EpiDermE, UPE-Université Paris-Est, Créteil, France; 8 CHU H Mondor, APHP, Créteil, France

**Keywords:** Burden, Albinism, Real life, Quality of life

## Abstract

**Background:**

Albinism comprises a group of autosomal recessive diseases that are characterized by poor vision and a variable hypopigmentation phenotype. A comprehensive literature review showed that no tool can assess the burden experienced by individuals who present with albinism, although such a tool is needed and would be beneficial for clinicians and patients alike.

**Method:**

The questionnaire was devised using standardized methodology for developing and validating questionnaires on the quality of life of subjects according to the following chronological structure: conceptual phase, development phase, and then validation phase. A multidisciplinary working group was assembled, including experts on questionnaire design and development, dermatologists specializing in care for patients with albinism, and representatives of the Genespoir association.

**Results:**

Based on an initial verbatim report, the workgroup compiled a list of items that were transcribed and reformulated into questions. During the validation phase, principal component analysis (PCA) was conducted on the 24 items, which allowed the questionnaire to be reduced to 20 questions [Q]. The standardized regression coefficients were all greater than 0.5 for their corresponding factors. Based on their normalized regression coefficients, each group of questions was linked to one of the following four dimensions, with each dimension consisting of at least three questions: “Live with” (8 Q), “Daily life” (3 Q), “Resignation” (3 Q), and “Fear of the future” (6 Q). All dimensions correlated well with the overall BoA score. Cronbach’s α was 0.92 for the entire BoA scale, confirming excellent internal coherence. Intradimensional coherences all demonstrated excellent reliability (α > 0.65). The BoA questionnaire was highly correlated with the SF12, RSES and DLQI validated questionnaires. This outcome confirmed the external validity.

**Conclusion:**

This questionnaire represents the first specific assessment tool for evaluating the burden of albinism. It is easy to use and relatively quick to complete, which will allow the burden to be evaluated over time with a reproducible questionnaire. To ensure that this questionnaire can be used by as many people as possible, cultural and linguistic validation in US English was conducted with the original French version.

**Electronic supplementary material:**

The online version of this article (10.1186/s13023-018-0894-3) contains supplementary material, which is available to authorized users.

## Background

Albinisms comprise a group of autosomal recessive diseases characterized by poor vision and a variable hypopigmentation phenotype. The prevalence of all known forms of albinism is 1:17000 newborns (range 1:10000–20,000) [1–7]. Different frequencies of several types of albinism have been reported in Asia [8–12], whereas the highest prevalence is found in some countries in Africa, mostly due to consanguinity issues and founder effects [13–15].

Albinism is associated with a defect in melanin biosynthesis responsible for the reduction of pigmentation in the skin, hair and eyes and for the visual defects. There are at least six types of non-syndromic OCA, named OCA1–6. Moreover, less common syndromic forms of albinism, such as Hermansky-Pudlak Syndrome (HPS) and Chediak–Higashi Syndrome (CHS), are characterized by more severe phenotypes, such as interstitial lung fibrosis, granulomatous colitis, bleeding problems and an increased susceptibility to bacterial infections beyond hypopigmentation and visual defects [12].

The characteristic ocular findings include various degrees of congenital nystagmus, hypopigmentation of the iris leading to iris translucency, reduced pigmentation of the retinal pigment epithelium, foveal hypoplasia, reduced visual acuity, and refractive errors. A degree of color vision impairment is found in different albinism types [16, 17]. Due to this visual deficiency, the autonomy of patients is often limited, thus impacting social and professional quality of life (QoL) [18]. Vision-specific QoL impairment in albinism was assessed by Kutzbach et al. in a comprehensive 2009 paper using a self-reported questionnaire on vision-related QoL. In this study, most notable impairment was recorded for distance acuity, vision-specific mental health, and vision-specific role difficulties [18].

The concept of “burden” has played an increasingly important role in evaluating the care of patients with chronic diseases, specifically skin diseases [19]. The term “global burden” was introduced by the WHO and has proven useful to quantify population health, thereby determining the priorities of action in the public health domain (WHO, www.who.int/topics/global_burden_of_disease/en/). In a recent study, Hay et al. [20] estimated the global burden of 15 skin diseases in 187 countries. The notion of burden has recently been extended to individuals and their families to assess disability, in its broadest sense, and physical aspects, related to various diseases, including infantile hemangioma [21], inherited ichthyosis [22], atopic dermatitis [23], and vitiligo [24]. However, there is currently no specific tool to measure the burden of albinism.

As part of its research activities, the reference center for rare skin disorder network, a French initiative, has implemented a 5-year cohort study to evaluate the burden of rare skin disorders, including albinism, in patients and their families. This burden must take into account not only health-related QoL but also social integration, emotional state, everyday life organization, and the use of medical resources, including consultations and medication. This initiative thus aimed to develop and validate an albinism-specific burden questionnaire, termed the Burden of Albinism (BoA).

## Methods

The self-administered BoA questionnaire was elaborated using standard methodology with three distinct phases: conceptual phase, developmental phase, and validation phase, with each phase following a well-defined process [25–27].

The questionnaire was conventionally built in a question/answer format. Response modalities were determined via expert consensus, and took the form of a 7-point Likert scale: “never” (0), “rarely” (1), “sometimes” (2), “often” (3), “very often” (4), and “constantly” (5). The answer "not concerned" is rated "0". Most of the questions included the wording “skin problem.”

### Conceptual phase

The initial conceptual phase involved 18 patients suffering from albinism who discussed their complaints and distress related to their condition. These verbatim transcripts were strengthened by a multidisciplinary working group comprising two dermatologists as well as an expert in the development of questionnaires. In the qualitative interviews, the primary fields reported by patients were the following: (1) the feeling of being discouraged by the condition, (2) changes in physical appearance, (3) fear of the future, (4) difficulty in initiating intimate relationships, (5) a general feeling of unease, and (6) the financial burden related to the disease. At this stage, 65 items were produced; reorganization and grouping of their content eventually resulted in 24 items.

### Development phase and validation phase

During this phase, the conceptual questionnaire was administered to a random sample of patients with albinism who attended consultation between July and November 2017, which was followed by an exploratory factor analysis in order to reveal latent constructs, assigning each item to its respective domain or dimension.

Principal component analysis was then performed in order to determine to which domain or dimension each question belonged, with varimax orthogonal rotation carried out. Whenever questions could be linked to several dimensions, the questions were allocated to the dimension deemed to be the most relevant semantically by the expert working group.

### Internal validity

To evaluate the questionnaire’s internal consistency, the homogeneity of the items in each dimension was tested using Cronbach’s alpha coefficient [28]. By this means, scores in the higher ranges like those above 0.7 generally suggest that the items are measuring the same entity, indicating good homogeneity.

To demonstrate the questionnaire’s unidimensionality, a higher order factor confirmatory analysis was performed aimed to confirm that the dimensions could be combined into one single score. The model’s goodness-of-fit was assessed using several criteria, namely the Bentler comparative fit index and Bentler-Bonett non-normed fit index. The criteria for a model’s goodness-of-fit were defined as a Bentler comparative fit index >0.90 and Bentler-Bonett non-normed fit index >0.90 [29]. The root mean square error of approximation (RMSEA) had to be around 0.05 or at the very least <0.08, with 0.05 contained within the confidence interval.

### External validity

To assess the questionnaire’s external validity, all participants were asked to complete three previously published and validated self-administered questionnaires: the SF12 quality of life questionnaire, Rosenberg Self-Esteem Scale (RSES), and Dermatology Life Quality Index (DLQI) questionnaire.

The SF12 is a short version of the SF-36, which is a well-known quality of life tool [30]. Based on 12 questions, a physical composite score (PCS) and mental composite score (MCS) are calculated.

The RSES is a widely used instrument that has been tested for reliability and validity in many settings [31]. The RSES instrument uses a 5-point scale that ranges from strongly agree to disagree to rate a series of 10 statements. The total score ranges from 0 to 30. Scores less than 15 suggest low self-esteem, while scores of ≥15 indicate normal self-esteem.

The DLQI questionnaire is the first dermatology-specific QoL instrument designed to assess the impact of skin diseases and associated treatments on patient QoL [32, 33]. Intended for patients aged >16 years, the DLQI represents the sum of all scores (0–30). The results can equally be expressed as percentages (0–100%).

Pearson correlation was calculated to assess the validity between BoA and the other three questionnaires. The data were analysed using SAS software Version 9.4 (SAS Institute, Cary, NC, USA) for Windows, with a significance level set at 0.05.

### Test-retest analysis

To assess reproducibility, a test-retest analysis was conducted. A group of subjects was asked to complete the questionnaire twice with a 10-day interval in-between.

### Translation, cross-cultural adaptation, and cognitive debriefing

Previously-validated methodology was applied to generate an US English-language version. This rigorous process comprising a meticulous 9-step procedure was meant to refine the translation while taking into account subtle nuances of the source document [34]. The different step employed to this end have been summarized in Table 1.

**Table 1:** Steps in linguistic and cultural validation

Stage	Details
Preparation	Evaluation of the source text from a linguistic and cultural point view including definition of concepts
Forward translations	Forward translation into the required target language by two independent translators
Reconciliation	Comparison of the two forward translations to provide the best adaption and produce a draft version of the text
Back translation	Translation of the draft forward translation back into the targeted language without reference to the original language
Back-translation review	Comparison of the original text and the back translation to verify that the meaning of the draft translation is equivalent to source
Analysis and implementation of back-translation review report	Analysis of the back-translation review report to verify if there are changes required to the draft forward
Pilot testing	Clinical review and cognitive debriefing
Review of cognitive debriefing or clinical review results	Review of the results from the cognitive debriefing or clinical review to identify translation modifications necessary for improvement

## Results

### Conceptual phase

The conceptual phase involved 18 patients who discussed their complaints and distresses related to albinism. This research resulted in an initial verbatim. The verbatim transcripts were strengthened by the comments of two dermatologists involved in an albinism clinic and day hospital and an expert in the design of burden questionnaires. A re-reading of these transcripts was performed by members of the patient support group. At that stage, 21 items formed the conceptual questionnaire (Table 2).

**Table 2:**
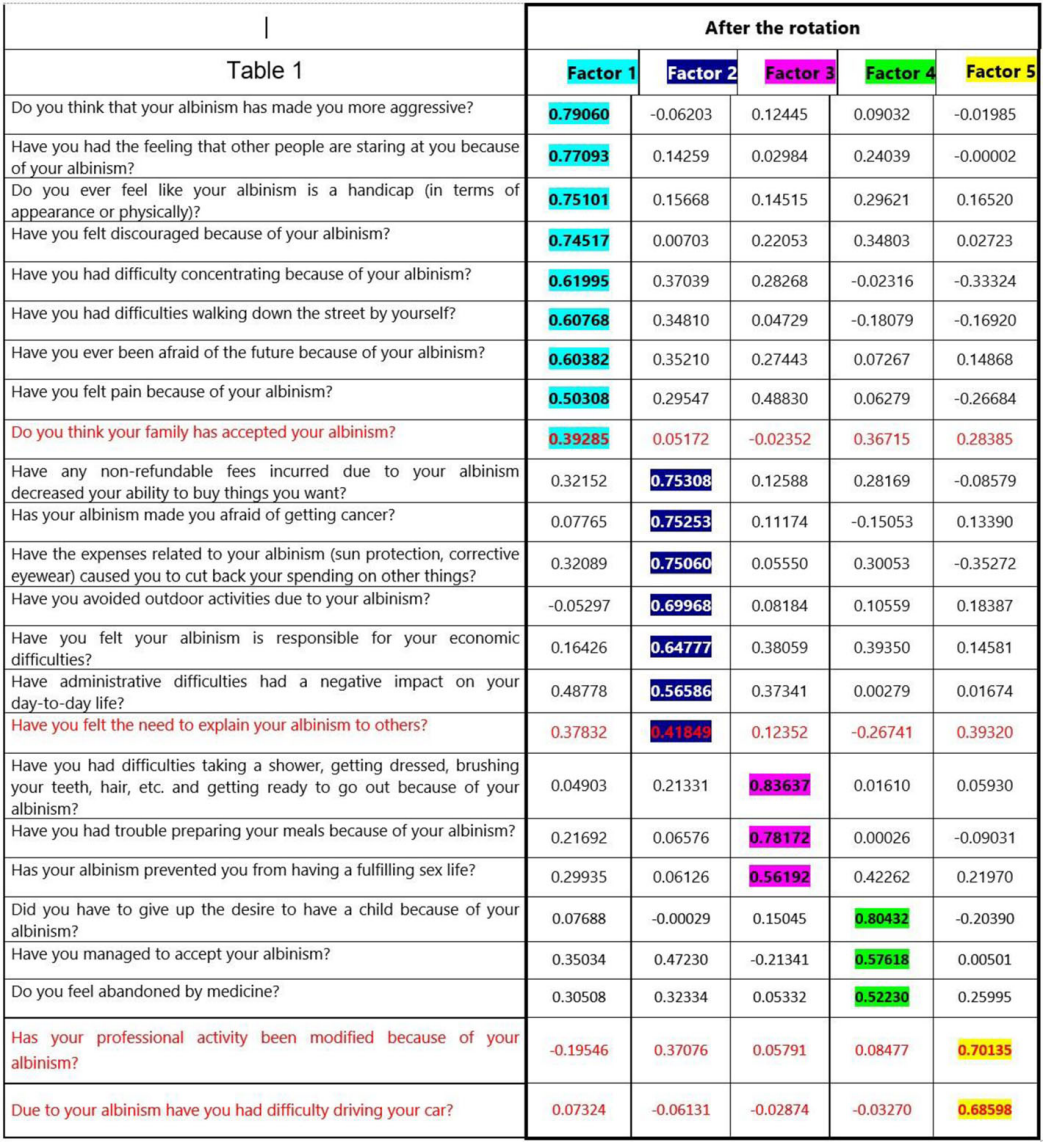
Standardized regression coefficients from the final rotated factor pattern

### Development and validation phase

#### Description of the study population

Overall, 87 patients who attended the clinic between July and November 2017 were invited, of which 63 agreed to participate to the study. Of these latter, 58% were female and 42% were male. The mean age of the participants was 44 ± 19.4 years (range: 38.7–49.25). No significant gender difference was observed (*p* = 0.13). Some 46.2% of the men and 52.8% of the women were employed. Regarding the delay before a correct diagnosis was established, 23.8% of the participants declared they felt the time to obtaining the correct diagnosis was delayed because of the physician who first examined them (46%) or due to tardy genetic diagnosis (33%). Of note, almost one out of five patients did not answer this question. More than two-thirds of the patients considered their albinism a disability (69.8%), whereas only 15.4% males and 16.7% females acknowledged that they were offered psychological follow-up.

Some 50% males and 52% females declared their albinism to be highly stigmatizing. Overall, 11.5% males and 33.3% females acknowledged difficulties in convincing their relatives of the handicap generated by their albinism. Similarly, among those engaged in a professional activity, 33.3 and 52.6% of the men and women, respectively, experienced difficulties in convincing their professional community of the handicap associated with their albinism.

### Internal validity

Principal component factor exploratory analysis was conducted on the entire cohort to test the robustness of the 24-item questionnaire, with standardized regression coefficients all greater than 0.5. Therefore, each group of questions was assigned a dimension. Five dimensions were highlighted, which were expressed in the form of a domain after the questionnaire was validated (Table 2):

- 1st Dimension with nine questions regarding resentment of the disease;
- 2nd Dimension with seven questions regarding disease-related expenses and plans for the future;
- 3rd Dimension with three questions regarding everyday life;
- 4th Dimension with three questions regarding acceptance and resignation;
- 5th Dimension with two questions regarding professional activity.

The fifth Dimension consisted of two questions: “Has your professional activity been modified because of your albinism?” and “Due to your albinism, have you had difficulty driving your car?” This fifth factor proved non-significant upon confirmatory analysis and was removed.

Two questions associated with Dimensions 1 and 2, namely, “Do you think your family has accepted your albinism?” and “Have you felt the need to explain your albinism to others?” had a coefficient <0.5. Their withdrawal proved legitimate as it did not alter the questionnaire’s robustness. Indeed, the percentage of explained variance associated with these two questions was low on confirmatory analysis (0.1646 and 0.1363, respectively), whereas the Cronbach’s α coefficients of the dimensions in which they were integrated increased when they were suppressed. After removing these questions, the BoA questionnaire was eventually structured around 20 questions classified into four dimensions.

The unidimensionality of the questionnaire was confirmed by higher order factor analysis. The practical indices of fit, namely the comparative fit index and non-normed fit index, proved to be acceptable (0.9163 and 0.9147, respectively; RMSEA = 0.0443; CI_90%_[0.0000; 0.0816]). Given the different indicators, the model was well adjusted, meaning that the three dimensions can be grouped into the same overall score.

Each group of questions was assigned a dimension, namely Living with albinism (8 items), Daily life (3 items), Resiliency (3 items), and Fear of the future (6 items), all dimensions correlating well with the overall BoA score. Cronbach’s α was 0.92 for the entire BoA scale, confirming its excellent internal coherence. Intradimensional coherences all demonstrated good reliability (α > 0.65).

### External validity

The concurrent external validity results were detailed in Table 3. The BoA questionnaire highly correlated with the SF12, RSES, and DLQI validated questionnaires, confirming the questionnaire’s external validity (Table 4).

**Table 3:** Correlations between the different scores

	PCS	MCS	DLQI	RSES	Global Score
PCS	1.00000	0.06986	−0.56161	0.17791	−0.52808
		0.6562	0.0002	0.2721	0.0007
	43	43	40	40	38
MCS	0.06986	1.00000	−0.48964	0.55391	−0.54514
	0.6562		0.0013	0.0002	0.0004
	43	43	40	40	38
DLQI	−0.56161	−0.48964	1.00000	−0.48176	0.67974
	0.0002	0.0013		0.0008	<.0001
	40	40	46	45	43
RSES	0.17791	0.55391	−0.48176	1.00000	−0.61403
	0.2721	0.0002	0.0008		<.0001
	40	40	45	45	42
Global Score	−0.52808	−0.54514	0.67974	−0.61403	1.00000
	0.0007	0.0004	<.0001	<.0001	
	38	38	43	42	44

*PCS* Physical Composite Score of SF12, *MCS* Mental Composite Score of SF12, *DLQI* Daily Life Quality Index Questionnaire, *RSES* Rosenberg’s Self-Esteem Scale

**Table 4:** Mean scores of the BoA questionnaire according to specific situations

	Yes	No	*p* value
Delay of diagnosis	46.61 ± 18.60	24.16 ± 13.66	<0.0001
Significant alteration in visual acuity	36 ± 12.57	24.25 ± 21.81	<0.0145
Do not talk about their albinism	40.07 ± 20.35	28.7 ± 13.34	<0.0243

### Cognitive debriefing, translation and cross-cultural adaptation

Cognitive debriefing did not result in any changes as to the wording of the questions. The original French version of the BoA questionnaire was translated and underwent linguistic and cultural validation in English (US). Both questionnaires are available as supplementary documentation (Additional file 1: Table S1).

### Scoring

The BoA questionnaire is composed of 20 questions, each scored from 0 to 5, which can be used and reported as a total score (range 0–100), where 0 indicates no impact, and 100 indicates maximal impact.

The total score is calculated by adding the scores from all 20 questions. The question “Have you managed to accept your albinism?” is scored in an inverse manner, namely, “never” is rated 5, “rarely” is rated 4, “sometimes” is rated 3, “often” is rated 2, “very often” is rated 1 and “constantly” is rated 0. The answer “not concerned” is rated zero.

### Sensitivity of the questionnaire

To assess the sensitivity of the BoA questionnaire, three subgroups were identified:

- Patients who had undergone a delay in diagnosis
- Patients with an important visual impairment that hinders their daily activities
- Patients who talk about their albinism versus those who do not speak about it

BoA scores for each of these situations confirm the questionnaire’s sensitivity. Patients who had experienced delayed diagnosis had a significantly higher burden than those who had not. Patients with significantly altered visual activity exhibited a significantly higher burden than those without. Patients who did not talk about their albinism had a significantly higher burden. The higher burden scores observed in those subgroups do, in fact, support the BoA questionnaire’s sensitivity.

## Discussion

Disease “burden” is increasingly being reported in the medical field in the care of chronic diseases and, more specifically, skin diseases [19]. The notion of global burden was introduced by the WHO. Different health authorities (NICE in the United Kingdom, HMO in the United States, and HAS in France) take into account the individual disease burden to determine reimbursement levels for medical products. Individual burden accounts for the broadest aspects of disease-related disability, including psychological, physical, social, and economic factors, that may aid in the development of specific care and management [22–25]. To the best of our knowledge, the BoA is the first specific tool that permits the assessment of the burden of albinism in adults.

The questionnaire is short (20 questions), understandable and easy-to-use by all patients.

The preliminary validation of the BoA has been established in the current study. BoA subscales were found to be psychometrically robust, with excellent internal consistency and good item-scale, convergent, and construct validity. The burden scores obtained with our new tool proved to be higher than those obtained with a non-specific instrument. In our view, this means that our new tool is better able to assess burden in its broadest sense, including aspects that are not taken into account by a non-specific QoL tools.

Patients with albinism experience difficulties mainly related to visual defects, including refractive errors, photophobia, nystagmus, and foveal hypoplasia [18]. Studies in different groups of patients with different degrees of visual deficiency could reveal additional factors that could impact QoL.

Patients with albinism generally have fair skin, a high incidence of sunburn, and early photoaging, including elastosis, actinic keratosis and skin cancer. A higher frequency of squamous cell carcinoma than basal cell carcinoma is observed among individuals with albinism [3]. Patients with many skin carcinomas or related complications may have a more severely altered QoL, especially those who live in very sunny regions and who have not been informed about the benefits of sun protection. Photoprotection may help prevent the early development of multiple, aggressive skin cancers that may occur in young adults.

The psychological impact of albinism may not only be related to visual alterations or skin lesions but also to the physical appearance associated with skin hypopigmentation, which may be different from that in other family members. Difficulty with intimate relationships and a general feeling of faintness may be present. Financial aspects related to treatment also appear to be recurrent complaints in patients with albinism.

A recent Brazilian study on QoL in patients with albinism described alterations in QoL from the physical point of view in a group of patients with albinism [35]. Skin lesions and a decrease in visual acuity were shown to impact QoL. Problems in personal, family and work relationships were often mentioned. Nevertheless, these complaints did not lead to alterations in the analysis of the social domain compared with the results of the control group. The questionnaire used in this study may not have been sensitive enough to detect alterations in QoL, given that it a generic questionnaire. Additionally, the group assessed in the study was heterogeneous and small-sized. According to the authors, more homogeneous groups are required to reveal the medical and social needs of patients with albinism [35].

Using the BoA questionnaire will facilitate the performance of the studies required to implement better health care for patients with albinism. Psychosocial aspects can be considered in addition to solar protection and skin lesion management. Family, genetic and professional guidance can be provided, such as participation in night labor activities, in addition to information promotion and demystification of the disease.

Our study exhibits several limitations. A first limitation of this research is related to the non-random patient selection, owing to the difficulty in recruiting patients in the context of a rare disease, although the number of patients recruited proves remarkable, resulting in an acceptance rate of rate 72%. Another limitation is that, owing to a lower self-perceived HRQL, the participants may have been more motivated to participate.

This questionnaire will be administered every 6 months to the patients included in the national RADICO-Fard cohort study. The objective is to obtain a 5-year follow up of patients with albinism treated by the national centers of reference and competence throughout the country. The main goal of this study, organized in France by INSERM and directed by the French network of rare skin disorders (FIMARAD), is to evaluate the individual burden of rare skin diseases, including albinism.

## Conclusion

In conclusion, the individual burden of patients with albinism must be recognized by physicians and carefully evaluated. The BoA questionnaire is, to the best of our knowledge, the first specific questionnaire dedicated to albino patients. Such questionnaire is highly needed and will help facilitate the implementation of better health care for these patients, taking into account the psychosocial aspects of disease-related disability, including psychological, physical, social, and economic factors.

## Additional file

Additional file 1: French and English (US) versions of the BoA questionnaire. (XLSX 10 kb)

## Abbreviations

BoA: Burden Of Albinism
CHS: Chediak–Higashi Syndrome
DLQI: Daily Life Quality Index Questionnaire
HPS: Hermansky-Pudlak Syndrome
OCA: Oculocutaneous Albinism
PCA: Principal Component Analysis
Qol: Quality Of Life
RSES: Rosenberg’s Self-Esteem Scale
SF12: Short Form 12
